# Adipose-Derived Stem Cells Cocultured with Chondrocytes Promote the Proliferation of Chondrocytes

**DOI:** 10.1155/2017/1709582

**Published:** 2017-01-04

**Authors:** Jie Shi, Jiulong Liang, Bingyu Guo, Yu Zhang, Qiang Hui, Peng Chang, Kai Tao

**Affiliations:** Reconstructive and Plastic Surgery, The General Hospital of Shenyang Military Region, Shenyang, China

## Abstract

Articular cartilage injury and defect caused by trauma and chronic osteoarthritis vascularity are very common, while the repair of injured cartilage remains a great challenge due to its limited healing capacity. Stem cell-based tissue engineering provides a promising treatment option for injured articular cartilage because of the cells potential for multiple differentiations. However, its application has been largely limited by stem cell type, number, source, proliferation, and differentiation. We hypothesized that (1) adipose-derived stem cells are ideal seed cells for articular cartilage repair because of their accessibility and abundance and (2) the microenvironment of articular cartilage could induce adipose-derived stem cells (ADSCs) to differentiate into chondrocytes. In order to test our hypotheses, we isolated stem cells from rabbit adipose tissues and cocultured these ADSCs with rabbit articular cartilage chondrocytes. We found that when ADSCs were cocultured with chondrocytes, the proliferation of articular cartilage chondrocytes was promoted, the apoptosis of chondrocytes was inhibited, and the osteogenic and chondrogenic differentiation of ADSCs was enhanced. The study on the mechanism of this coculture system indicated that the role of this coculture system is similar to the function of TGF-*β*1 in the promotion of chondrocytes.

## 1. Introduction

Articular cartilage plays an important role in joint activities. Articular cartilage is devoid of blood vessels, lymphatics, and nerves while it is subject to harsh biomechanical environments. With the development of aging population and the increase of high energy and high speed trauma, the patients with articular cartilage injury and degeneration are increasing [1]. Articular cartilage is mainly formed by chondrocytes and an abundant extracellular matrix (ECM), composed of collagen and proteoglycan. The chondrocyte is the resident cell type in articular cartilage. Chondrocytes are highly specialized, metabolically active cells that play a unique role in the development, maintenance, and repair of the ECM. Unfortunately, chondrocytes have limited potential for replication, a factor that contributes to the limited intrinsic healing capacity of cartilage in response to injury. Therefore, its ability to repair itself is very finite [2].

Articular cartilage injury can be divided into partial defect of the full thickness of the cartilage and full defect of the full thickness of the cartilage according to the degree of injury. When the articular cartilage injury exceeds the body's ability to repair itself, damage area will be replaced by fibrocartilaginous tissue, which is different in biochemical composition and biomechanical properties when compared with articular cartilage. Fibrocartilaginous tissue cannot meet the needs of the joint exercise, which leads to the scope of the lesion expanding causing arthritis and leading to intractable pain and dysfunction. Therefore, the repair of articular cartilage injury has always been a difficult and hot spot in the field of orthopedic research [3–6].

The treatment of injured cartilage commonly used today cannot really restore the organizational structure and biomechanical property of cartilage. Tissue engineering is the development of the alternates to replace or support the function of defective or injured body parts using the principle of cell biology and engineering. It provides a new concept and technique to deal with tissue defect and functional disability.

As the primary factor in cartilage tissue engineering, the selection and acquisition of seed cells is most important. Autologous chondrocyte is the only seed cell which has been used in clinics, but its application is limited because of the donor site mobility and difficulty in isolation and cultivation. Adult stem cells are widespread in the body, especially adipose-derived stem cells (ADSCs). ADSCs are ideal seed cells for tissue engineering because of their accessibility and abundance.

Traditional methods used to induce ADSCs to differentiate into chondrocytes require a large number of cytokines, which are expensive and cannot be widely used in clinical practice. Several studies have found that the microenvironment of articular cartilage can induce ADSCs to differentiate into chondrocytes, but whether the fat stem cells cocultured with articular cartilage can promote the growth of chondrocytes has not been studied.

ADSCs are adult stem cells originating from the mesoderm and have become an ideal source of seed cells and are search hotspot. ADSCs are a convenient, easily obtainable, adequate source of stem cells with only minimal injury to the body as compared to other adult stem cell sources with similar biological characteristics and differentiation potentials such as bone-marrow mesenchyme stem cells (BMSCs). Not only are ADSCs easier to obtain in adequate quantities but also they are offering better proliferative capacity when compared to BMSCs.

Transforming growth factor-*β*1 (TGF-*β*1) is a multifunctional regulator of cell growth and extracellular matrix synthesis; it can promote the proliferation of chondrocytes, increase synthesis of both proteoglycan and type II collagen, and reduce the expression and secretion of the cartilage collagenase. Moreover TGF-*β*1 could inhibit inflammatory response and promote tissue inhibitor of metalloproteinase expression [7].

In our present study, when ADSCs and chondrocytes were cocultured, we found the proliferation of chondrocytes was promoted and the apoptosis of chondrocytes was inhibited. This coculture system promoted chondrocytes to produce more anabolic proteins and inhibit catabolic proteins. We also found that these phenomena are closely related to the TGF-*β*1 pathway. The findings indicated that coculture of ADSCs and chondrocytes can stimulate the promoting effects of TGF-*β*1, which may provide a new solution for the treatment of cartilage injury.

## 2. Materials and Methods

### 2.1. Isolation of Rabbit ADSCs

ADSCs were isolated from rabbit adipose tissue according to published protocol [8, 9]. Briefly, the posterior cervical subcutaneous adipose tissue was harvested from the rabbits (skeletally mature female New Zealand white rabbits, weighing between 2.5 and 3.0 kg) and washed with PBS. The tissue samples were cut into small pieces and 2 g fat tissue was added in each 25 mL of 0.12% of collagenase-PBS solution (Invitrogen) to digest at 37°C for 45 min. The mixture was shaken every 15 min during the digestion. Immediately after the reaction was completed, 25 mL of Dulbecco's Modified Eagle Medium (DMEM) (Invitrogen) was added to neutralize collagenase activity. The resulting solution was filtered using a 70 *μ*m nylon membrane. The filtrate was centrifuged at 1300 rpm for 6 min at 25°C, and the supernatant was removed. The pellet of ADSCs was resuspended in DMEM containing 10% Fetal Bovine Serum (FBS) (Invitrogen) and seeded in 60 cm^2^ tissue culture dishes at the density of 5 × 10^4^ cells/cm^2^ and cultured with culture medium, DMEM containing 10% FBS (Invitrogen), 100 units/mL penicillin, and 100 *μ*g/mL streptomycin at 37°C, 5% CO_2_, and 95% air.

### 2.2. Purification and Characterization of ADSCs

The ADSCs were purified and characterized by an Epics XL flow cytometer and immune staining. For flow cytometry assay, the ADSCs were placed in a centrifuge tube (1 × 10^6^/tube) and incubated with 1 *μ*g of the following specific antibodies including mouse anti-CD90 antibody (http://www.antibodies-online.com/, Cat #ABIN118259), mouse anti-CD44 antibody (http://www.antibodies-online.com/, Cat #ABIN2226505), goat anti-CD73 antibody (Santa Cruz Biotechnology, Inc, Cat #sc14684), mouse anti-CD31 antibody (http://www.antibodies-online.com/, Cat #ABIN3024342), and mouse anti-CD45 antibody (http://www.antibodies-online.com/, Cat #ABIN1449134) over night at 4°C. The next morning, the cells in each sample were washed with PBS three times. They were then either incubated for 1 hour at 4°C with 1 *μ*g of FITC-conjugated goat anti-mouse IgG antibody (Thermo Fisher Scientific, Cat #F2761) to test for CD90, CD44, CD31, and CD45, or incubated with Cy3-conjugated donkey anti-goat IgG antibody (Abcam, Cat #ab6949) to test for CD73. For CD34 expression, the cells were incubated with 1% goat serum at room temperature for 30 min and reacted with 1 *μ*g of rabbit anti-CD34 antibody (Bioss Antibodies, Cat #bs-2038R) overnight at 4°C, then washed with PBS three times, and reacted with 1 *μ*g of Cy3-conjugated goat anti-rabbit IgG (Abcam, Cat #6939) for 1 hour at 4°C. The ADSCs incubated with PBS instead of primary antibodies were used as control samples. After washing three times with PBS, the samples were analyzed using an Epics XL flow cytometer and positively stained cells on CD90, CD44, and CD73 and negatively stained on CD31, CD34, and CD45 were collected for the following experiments.

For immune staining, the ADSCs were seeded in a 12-well plate at a density of 3 × 10^4^/well and cultured with DMEM-10% FBS for 3 days. The cells were washed once with PBS and fixed with PBS buffered 4% paraformaldehyde for 30 min. The fixed cells were incubated with primary antibodies including mouse anti-rabbit CD90, CD44, CD31, and CD45 antibodies (1 : 300 dilution) and goat anti-rabbit CD73 antibody (1 : 200 dilution) overnight at 4°C. For CD34 staining, the cells were incubated with 1% goat serum-PBS for 30 min and then reacted with rabbit anti-CD34 antibody (1 : 300 dilutions) at room temperature for 2 hours. The ADSCs incubated with PBS instead of primary antibodies were used as control samples. After the incubation with primary antibodies, all cells were washed with PBS three times and then reacted with Cy3-conjugated goat anti-rabbit IgG (1 : 500 dilutions) at room temperature for 1 hour. The fluorescent images of stained cells were obtained under an inverted fluorescent microscope (Nikon Eclipse) and analyzed by SPOT imaging software.

### 2.3. Isolation and Culture of Chondrocytes

Primary chondrocytes were isolated from female rabbit as previously described [10]. Briefly, slices of cartilage from the femoral head, femoral condyles, and tibial plateau then were stripped, diced, and digested twice in collagenase (3 mg/mL of collagenase for 1 h and then 0.5 mg/mL of collagenase overnight at 37°C). Undigested cartilage was removed by a 70 *μ*m nylon filter and the filtrate containing chondrocytes was centrifuged at 1200 rpm for 5 min, supernatant was discarded, and the pellet was resuspended in culture medium and cultured in an incubator with 95% air and 5% CO_2_ at 37°C and used in the following experiments. The ADSCs were cocultured with chondrocytes (1 : 1) in transwell chambers [11].

### 2.4. Cell Culture

**Figure 1:**
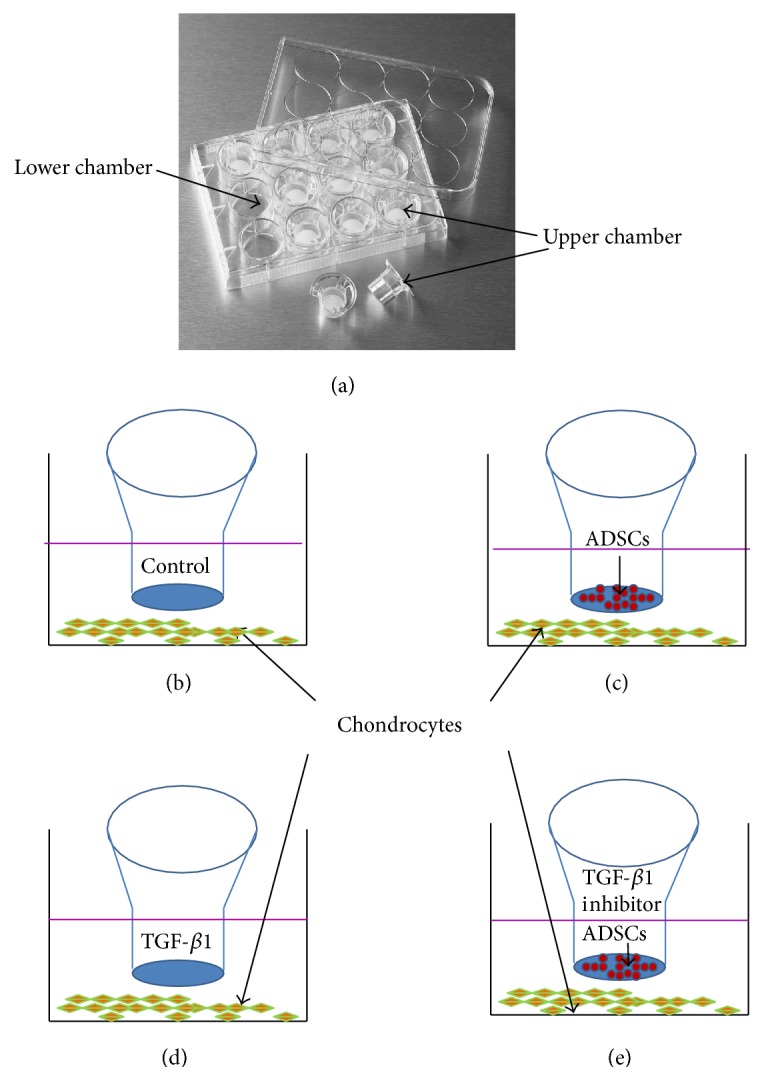
The experimental model. (a) The picture of transwell chamber. ((b)–(e)) The schematic diagram of the four groups, respectively, for chondrocytes cultured in normal culture medium (b), chondrocytes cocultured with ADSCs together (c), chondrocytes cultured with TGF-*β*1-containing medium (d), and chondrocytes cocultured with both ADSCs and TGF-*β*1 inhibitor (e).

In order to study the coculture effect of ADSCs on the proliferation of chondrocytes, we cultured chondrocytes using a novel transwell chamber (Figure 1(a)) with four different conditions up to 12 days. Group 1 (cartilage cell group): chondrocytes were seeded in the well directly (lower chamber) at the density of 1 × 10^5^/well in a 24-well plate (Figure 1(b)) and cultured with culture medium (DMEM-10% FBS). Group 2 (coculture group): chondrocytes were seeded in the well directly (lower chamber) at the density of 1 × 10^5^/well and ADSCs were seeded in the up-chamber at the density of 1 × 10^5^/well in a 24-well plate. The cells were cultured with culture medium (Figure 1(c)). Group 3 (TGF-*β*1 group): chondrocytes were seeded in the well directly (lower chamber) at the density of 1 × 10^5^/well in a 24-well plate and cultured in culture medium with 5 ng/mL of TGF-*β*1 (Figure 1(d), final concentration, R&D Systems, Minneapolis, MN). Group 4 (coculture + TGF-*β*1 inhibitor group): chondrocytes were seeded in the well directly (lower chamber) at the density of 1 × 10^5^/well and ADSCs were seeded in the up-chamber at the density of 1 × 10^5^/well in a 24-well plate. The cells were cultured in culture medium with 15 *μ*M of a TGF-*β*1 inhibitor (Galunisertib, LY2157299, Selleck, USA) (Figure 1(e)). The capability of cellular proliferation and differentiation was measured by morphology, MTT assay, and histochemical staining.

### 2.5. Apoptosis Analysis Using Hoechst 33258 Staining

The apoptosis of chondrocytes cultured in different conditions was analyzed by Hoechst 33258 staining. After being cultured for 5 days the cartilage cells were washed twice with PBS and incubated with 1 *μ*g/mL Hoechst 33258 for 5 min at room temperature. Cellular and nuclear morphology was examined using phase and fluorescence microcopy.

### 2.6. Cell Proliferation Assay

The proliferation of chondrocytes cultured in different conditions was determined by MTT assay. The cartilage cells were cultured in a 24-well plate with different conditions as described above for 0, 3, 6, 9, and 12 days. At the end of the each time point, each 50 *μ*L of 5 mg/mL of MTT was added to each well and incubated at 37°C for 4 hours. The cells were periodically viewed under an inverted microscope. When the purple precipitate was clearly visible under the microscope, 500 *μ*L of detergent reagent was added to each well and swirled gently. The plate was left in the dark for another 4 hours at room temperature, and then 200 *μ*L of the reaction mixture from each well of a 24-well plate was transferred into a new 96-well plate. The optical densities at 570 nm of the 96-well plates were measured using a microplate reader (BIO-RAD).

### 2.7. Histochemical Staining on Chondrocytes with Three Culture Conditions

After culturing with different conditions for 9 days cells were stained by alcian blue, toluidine blue, and safranin O through the protocols. Briefly, at the end of the culture, the medium was removed from each well and the cells were washed once with PBS. The cells were fixed with 10% neutral formalin in PBS for 20 min at room temperature and then rinsed 3 times with PBS. The fixed cells were treated with either alcian blue solution (1 g of alcian blue GX in 100 mL of 3% acetic acid) for 2 hours at room temperature, or 0.1% toluidine blue staining solution for 5 min, or 0.1% safranin O for 5 min. Then the cells were washed three times with water and the images of stained cells were analyzed under a microscope.

### 2.8. Glycosaminoglycan (GAG) Assay in the Culture Medium

GAG production in the medium of chondrocytes cultured with different conditions for 5 days was analyzed using GAG assay kit (Beyotime, China) according to the manufacturer's specifications [12]. All the measurements were normalized to total cell numbers in each culture condition.

### 2.9. Quantitative Real-Time Reverse Transcription Polymerase Chain Reaction (qRT-PCR)

Total RNA was isolated from chondrocytes after being cultured with different conditions for 5 days by TRIzol kit (Invitrogen) according to the manufacture's protocol [13]. Complementary DNA was synthesized by reverse transcription of total RNA using a RT reaction kit (Promega). Real-time PCR was performed using an Mx3000P real-time PCR system (Applied Biosystems) according to the manufacturer's instruction and SYBR Premix Ex Taq (TaKaRa) as a DNA-specific fluorescent dye. The primer sequences for detection of mRNA expression were shown in Table 1.

All the reactions were repeated at least three times. Gene expression levels were calculated relative to *β*-actin by using Stratagene Mx3000P software.

### 2.10. Western Blot Analyses

To determine the expression of protein in chondrocytes cultured under different conditions for 5 days, whole cell extracts (lysate) were prepared from 1 × 10^6^ cells in lysis buffer (Beyotime, China). The protein samples (30 *μ*g/each) were subjected to 12% SDS-polyacrylamide gels and transferred onto a nitrocellulose membrane. Target proteins were probed with specific antibodies—Cyclin D1, caspase-3, mmp3, mmp13, TGF-*β*1, SOX9, collagen type II alpha 1 chain (col2*α*1), and peroxisome proliferator-activated receptor-gamma coactivator-1 alpha (PGC-1*α*), and normalized with glyceraldehyde 3-phosphate dehydrogenase (GAPDH). All antibodies were obtained from Santa Cruz.

### 2.11. Statistical Analysis

All experiments were performed in triplicate and all data were analyzed by Student's* t*-test and presented as the mean ± SD. A *p* value < 0.05 was considered to be significant.

## 3. Results

### 3.1. Purification and Characterization of ADSCs

**Figure 2:**
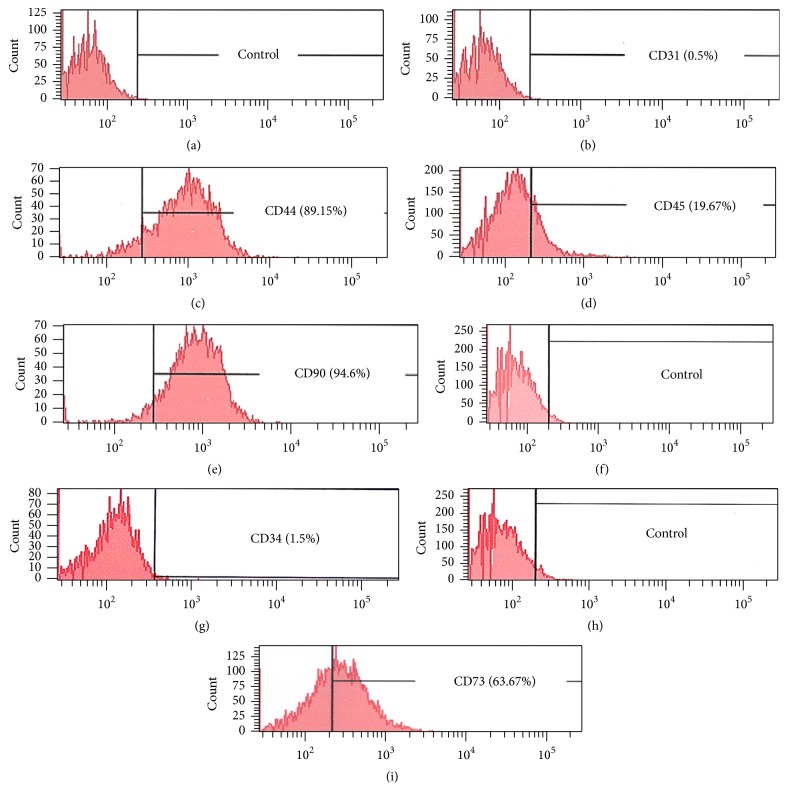
The characterization of ADSCs identified by flow cytometry. (a) FITC negative control result was obtained from ADSCs (1 × 10^6^) incubated with FITC-conjugated goat anti-mouse IgG antibody only. (b) CD31 expression in ADSCs incubated with CD31 antibody and detected by FITC-conjugated second antibody. (c) CD44 expression in ADSCs incubated with CD44 antibody and identified by FITC-conjugated second antibody. (d) CD45 expression in ADSCs incubated with CD45 antibody and tested by FITC-conjugated second antibody. (e) CD90 expression in ADSCs incubated with CD90 antibody and determined by FITC-conjugated second antibody. (f) Negative control result of ADSCs incubated with Cy3-conjugated goat anti-rabbit IgG antibody only. (g) CD34 expression in ADSCs incubated with CD34 antibody and identified by Cy3-conjugated goat anti-rabbit IgG antibody. (h) Negative control result of ADSCs incubated with Cy3-conjugated donkey anti-goat IgG antibody only. (i) CD73 expression in ADSCs incubated with anti-CD73 antibody and identified by Cy3-conjugated donkey anti-goat IgG antibody.

**Figure 3:**
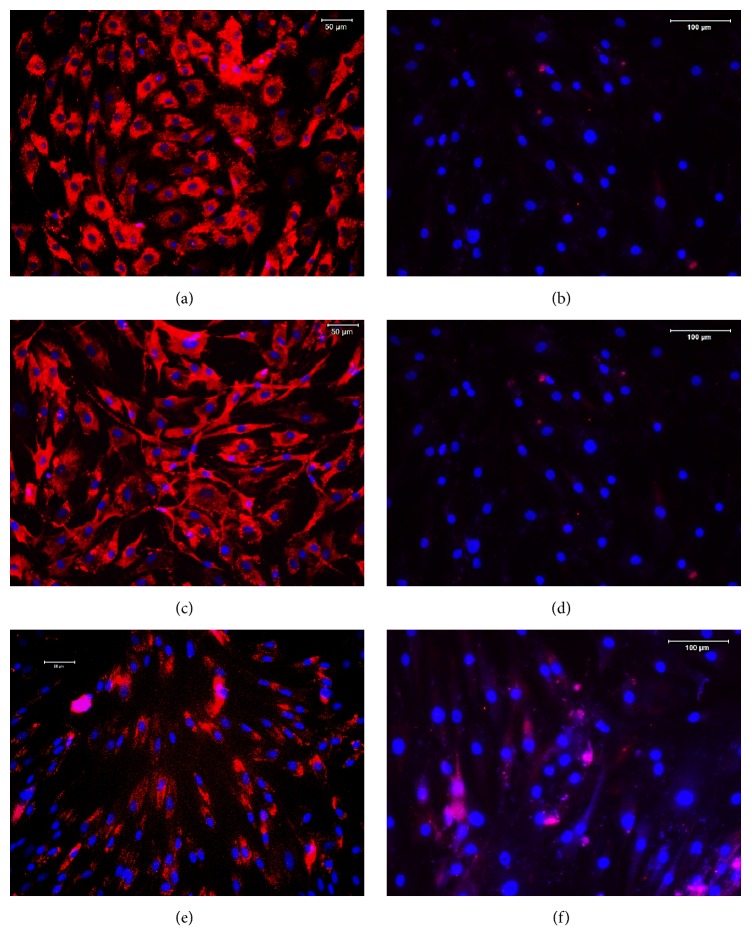
Stem cell marker expression in ADSCs. The stem cell markers in ADSCs were identified by specific antibody reactions with CD44 (a), CD31 (b), CD90 (c), CD34 (d), CD73 (e), and CD45 (f), respectively. More than 90% of ADSCs were positively stained by CD44 (a) and CD90 (c). However, less than 70% of ADSCs expressed CD73 (e), and less than 20% of ADSCs exhibited CD45 (f). On the other hand, very few ADSCs were positively stained by CD31 (b) and CD34 (d). The images were obtained by an inverted fluorescent microscope. Bars: 100 *μ*m.

The ADSCs were isolated from rabbit adipose tissues and purified by a flow cytometry. The immune staining and flow cytometry assay (FCA) showed that ADSCs isolated from rabbit adipose tissues expressed high levels of CD44 (89.15%, Figures 2(c) and 3(a)), CD90 (94.6.15%, Figures 2(e) and 3(c)), and CD73 (63.67%, Figures 2(i) and 3(e)). In contrast, only a small proportion of rabbit ADSCs exhibited the hematopoietic markers CD31 (0.5%, Figures 2(b) and 3(b)), CD34 (1.5%, Figures 2(g) and 3(d)), and CD45 (19.67%, Figures 2(d) and 3(f)). These findings indicated that ADSCs have been isolated from rabbit adipose tissues and can be used for further experiments.

### 3.2. ADSCs Promoted the Proliferation of Chondrocytes via TGF-*β*1 Pathway

**Figure 4:**
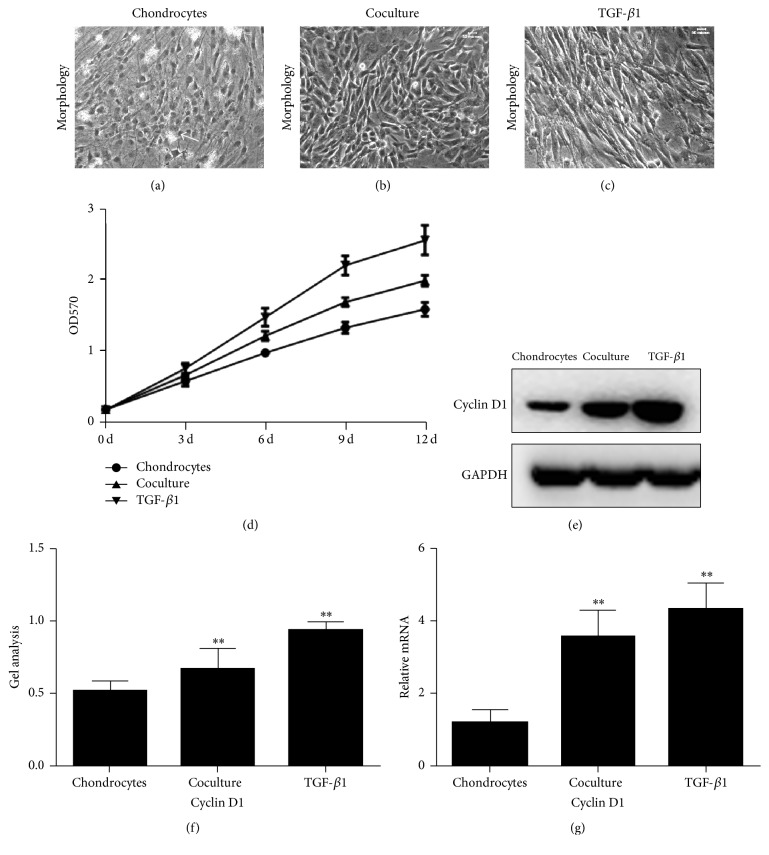
Proliferation of chondrocytes cultured with three different conditions for 5 days. Morphology of chondrocytes culture with a control medium (a), ADSCs coculture condition (b) and TGF-*β*1-containing medium (c). Growth curves of chondrocytes cultured in three different conditions are shown ((d), (e)). Chondrocytes: chondrocytes were cultured in normal culture medium. Coculture: chondrocytes were cocultured with ADSCs. TGF-*β*1: chondrocytes were cultured with TGF-*β*1-containing medium. All were determined by MTT assay. Cyclin D1 protein expression in chondrocytes cultured in three different conditions for 5 days as determined by western blot (f). Gene expression of Cyclin D1 in chondrocytes cultured in three different conditions for 5 days as determined by real-time PCR. Data are shown as mean ± SEM. ^*∗∗*^ *p* < 0.01 versus control group (chondrocytes).

In order to study the effect of ADSCs on the proliferation of chondrocytes, we isolated two different kinds of cells. ADSCs isolated from rabbit adipose tissues and chondrocytes from cartilage tissues. The mechanisms of the effect of ADSCs on the proliferation of chondrocytes were also studied. The chondrocytes isolated from rabbit cartilage were cultured with ADSCs together using a novel transwell chamber (Figure 1(a)). The morphology assay showed that chondrocytes cultured with normal culture medium (control group) for 5 days exhibited spindle shape with some apoptosis of cells (Figure 4(a)). When the chondrocytes were cultured with ADSCs, the chondrocytes were still in spindle shape with a decrease in the apoptosis of cells and an increase of cell numbers (Figure 4(b)). The addition of TGF-1*β* to the culture medium of chondrocytes increased the cell numbers significantly and largely inhibited the apoptosis of cells (Figure 4(c)). These results were further demonstrated by MTT assay. The proliferation of the chondrocytes was promoted when chondrocytes were cocultured with ADSCs (Figure 4(d)). Moreover, the chondrocytes grew much faster in 5 *μ*g/L of TGF-*β*1-containing medium than both control group (*p* < 0.05, Figure 4(d)) and ADSCs-chondrocytes coculture group (*p* < 0.05, Figure 4(d)). The proliferation rate of chondrocytes grown in three conditions was TGF-*β*1 > ADSCs > control.

These results were further confirmed by real-time PCR (Figure 4(g)) and western blot (Figures 4(e) and 4(f)). Gene expression of Cyclin D1 in TGF-1*β* group was found three times higher than that in control group (Figure 4(g)) and ADSCs-chondrocytes coculture group exhibited 1.98 times higher Cyclin D1 gene than that of control group (Figure 4(g)). Similarly, the protein expression of Cyclin D1 was upregulated in chondrocytes cultured with TGF-1*β* and ADSCs-chondrocytes coculture system (Figures 4(e) and 4(f)). The semiquantifying of results indicated that chondrocytes cocultured with ADSCs produced 30% more Cyclin D1 than control group and when cultured with TGF-*β*1 (Figures 4(e) and 4(f)) produced 82% more of Cyclin D1 than control group.

**Figure 5:**
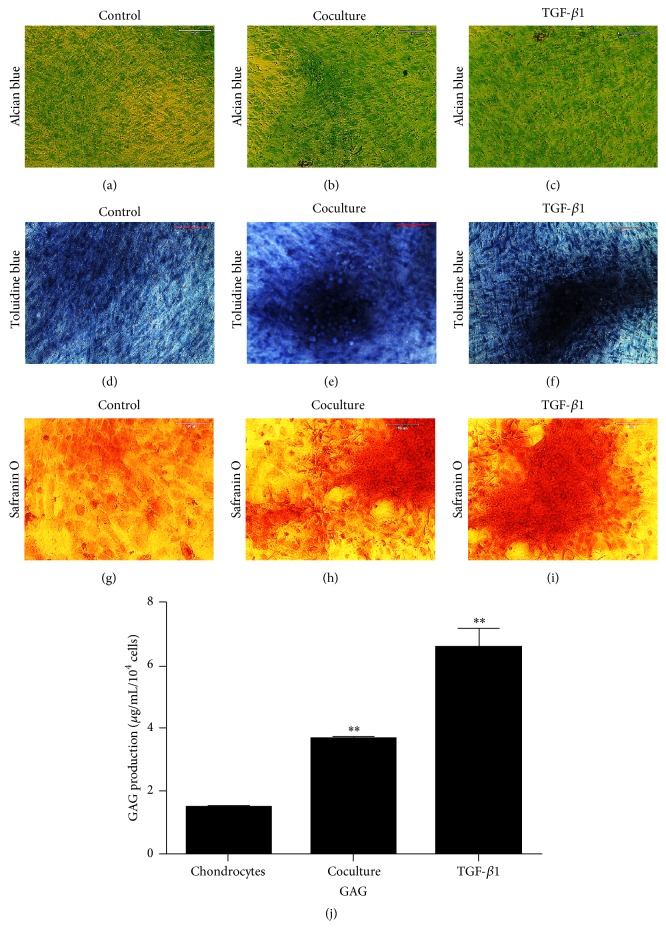
Chondrogenesis marker expression in chondrocytes cultured with three different conditions for 9 days detected by histochemical staining ((a)–(i)) and ELISA (j). Alcian blue staining is shown in ((a)–(c)). Toluidine blue staining is shown in ((d)–(f)). Safranin O staining is shown in ((g)–(i)). Chondrocytes were cultured in control medium ((a), (d), and (g)), or ADSCs cocultured system ((b), (e), and (h)), or TGF-*β*1-containing medium ((c), (f), and (i)) and stained by the above three markers. The production of glycosaminoglycan (GAG) was analyzed using ELISA kit (j). Data are shown as mean ± SEM. ^*∗∗*^ *p* < 0.01 versus control group. Control: chondrocytes were cultured in normal culture medium. Coculture: chondrocytes were cocultured with ADSCs together. TGF-*β*1: chondrocytes were cultured with TGF-*β*1-containing medium.

The coculture effect of ADSCs on the proliferation of chondrocytes was also investigated by histochemical staining of chondrocyte-related protein expressions. The results demonstrated that chondrocytes grew much faster in TGF-*β*1-containing medium (Figures 5(c), 5(f), and 5(i)) than those grown in ADSCs coculture condition (Figures 5(b), 5(e), and 5(h)) and control group (Figures 5(a), 5(d), and 5(g)).

After 5 days the cells that were cultured in the medium containing TGF-*β*1 were stained at a rate of over 95% with all three stains, alcian blue (Figure 5(c)), toluidine blue (Figure 5(f)), and safranin O (Figure 5(i)). Similarly cells cocultured with ADSCs were positively stained more than 85% of the time with alcian blue (Figure 5(b)), toluidine blue (Figure 5(e)), and safranin O (Figure 5(h)). The control group cells were positively stained about 70% of the time with each of the three stains alcian blue (Figure 5(a)), toluidine blue (Figure 5(d)), and safranin O (Figure 5(g)).

The production of glycosaminoglycan (GAG) in the medium of chondrocytes cultured under three different conditions was determined using an Elisa kit. The results showed that the chondrocytes produced more GAG when they were either cultured with TGF-*β*1-containing medium (3.36 times more than the control sample) or cocultured with ADSCs (1.42 times more than the control sample) (Figure 5(j)).

**Figure 6:**
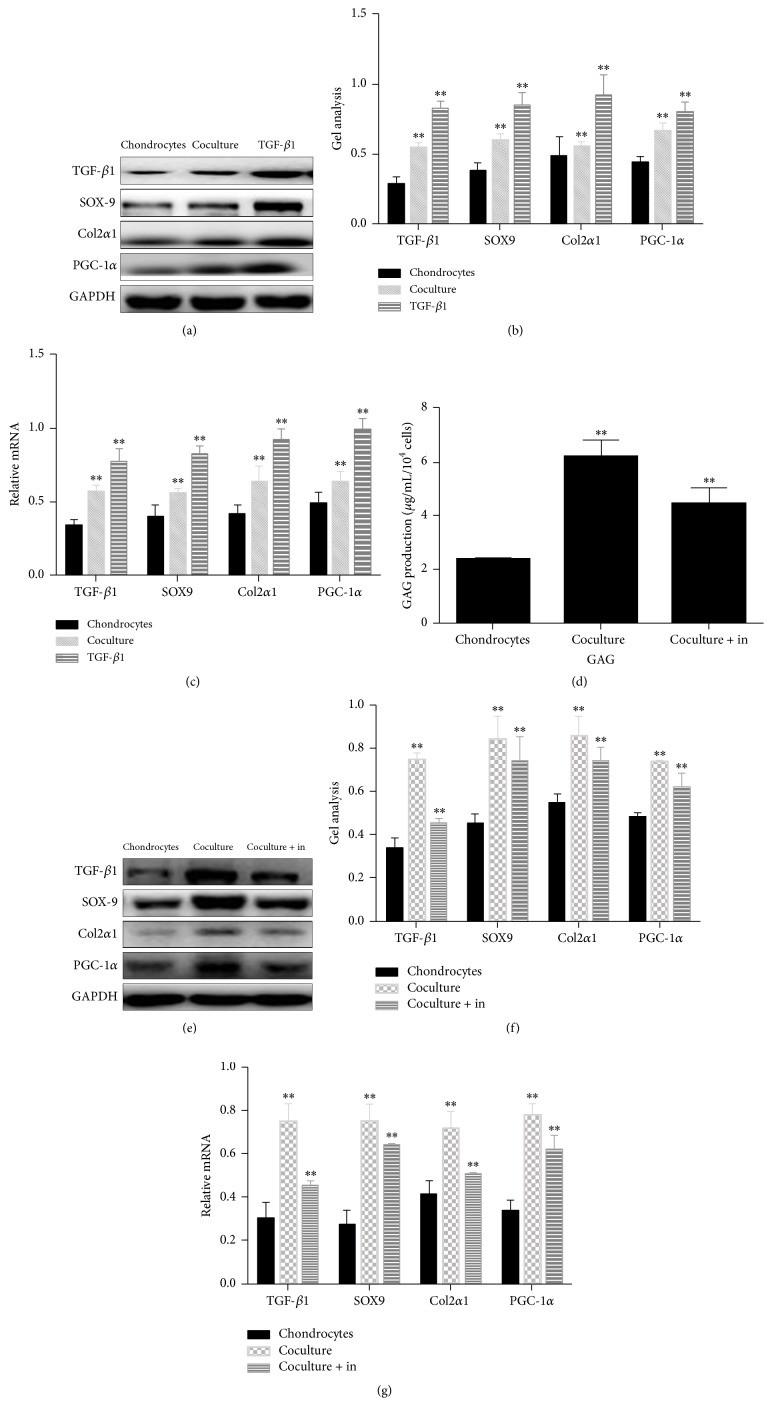
Mechanisms of promoting effect of ADSCs on chondrocytes investigated by western blot ((a), (b), (e), and (f)), real-time PCR ((c),(g)), and ELISA kit (d). Chondrocytes were cultured in control medium (chondrocytes), ADSCs cocultured system (coculture), TGF-*β*1-containing medium (TGF-*β*1), and ADSCs cocultured system plus TGF-*β*1 inhibitor (coculture + in) for 5 days. Both western blot ((a), (b), (e), and (f)) and real-time PCR ((c), (g)) results indicated that TGF-*β*1 level in chondrocytes either cocultured with ADSCs or cultured with TGF-*β*1-containing medium was upregulated. Furthermore, chondrocytes produced more the following proteins and genes including SOX-9, collagen type II (*α*1), and PGC-1*α* when they were cultured with either TGF-*β*1-containing medium or ADSCs cocultured system. In addition, the production of glycosaminoglycan (GAG) in chondrocytes cocultured with ADSCs was also increased. However, after adding TGF-*β*1 inhibitor to ADSCs cocultured system, the production of GAG in chondrocytes was decreased (d). Furthermore, all the above-mentioned proteins and genes were decreased in chondrocytes cultured with ADSCs plus TGF-*β*1 inhibitor ((e), (f) and (g)). Data are shown as mean ± SEM. ^*∗∗*^ *p* < 0.01 versus chondrocytes group.

Western blot results indicated that higher expression of TGF-*β*1 was found in the chondrocytes cultured with either TGF-*β*1-containing medium (Figures 6(a) and 6(b)) or coculture system (Figures 6(a) and 6(b)). Furthermore, three chondrocyte-related proteins, SOX-9, collagen type II (Col-2*α*1), and PGC-1*α*, were upregulated in the chondrocytes cultured with either TGF-*β*1-containing medium or ADSCs coculture system (Figures 6(a) and 6(b)). These findings were further confirmed by real-time PCR (Figure 6(c)). However, when we added Galunisertib, a TGF-*β*1 inhibitor, to the medium of ADSCs cocultured with chondrocytes, the amount of GAG released from chondrocytes was significantly reduced (Figure 6(d)). Furthermore, when the cells were cocultured with TGF-*β*1 inhibitor, TGF-*β*1 and all three chondrocyte-related proteins SOX9, Col2*α*1, and PGC-1*α* were downregulated (Figures 6(e), 6(f), and 6(g)).

### 3.3. ADSCs Inhibited the Apoptosis of Chondrocytes through TGF-*β*1 Pathway

**Figure 7:**
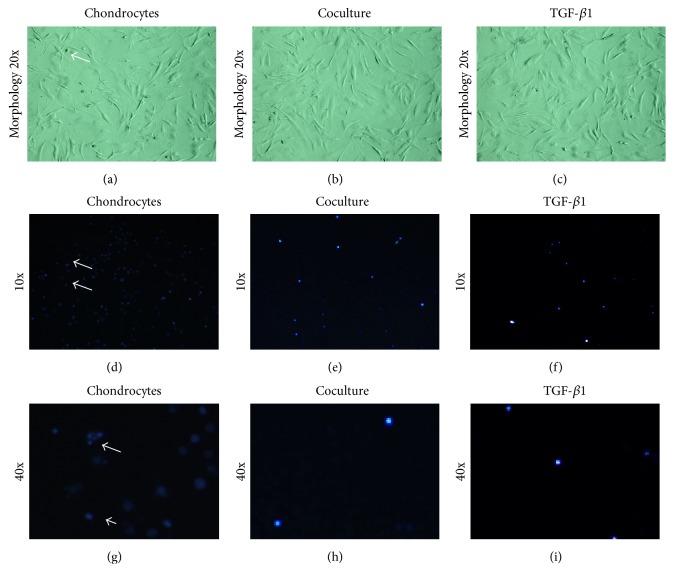
Morphology ((a)–(c)) and apoptosis assay ((d)–(i)) of chondrocytes cultured with control medium ((a), (d), and (g)), ADSCs cocultured system ((b), (e), and (h)), and TGF-*β*1-containing medium ((c), (f), and (i)) for 5 days. Morphology assay showed some membrane blebbing in the chondrocytes cultured with control medium (a), and the cell blebbing numbers were decreased in chondrocytes cultured with ADSCs cocultured system (b) and TGF-*β*1-containing medium (c). Hoechst 33258 staining showed many chromatin condensations in chondrocytes cultured with control medium ((d), (g)), and much less apoptosis of cells was found in chondrocytes cultured with ADSCs cocultured system ((e), (h)) and TGF-*β*1-containing medium ((f), (i)). The images of (g), (h), and (i) were the corresponding higher magnification images of (d), (e), and (f), respectively.

We next studied the effect of ADSCs cocultured with chondrocytes on cell apoptosis. Chondrocytes cultured with control medium showed much membrane blebbing as well as nuclear condensation and fragmentation (Figures 7(a), 7(d), and 7(g); see arrows). However, these apoptosis hallmark features were inhibited in chondrocytes cultured with ADSCs coculture group and 5 ng/mL of TGF-*β*1 culture group (Figures 7(e), 7(f), 7(h), and 7(i)).

**Figure 8:**
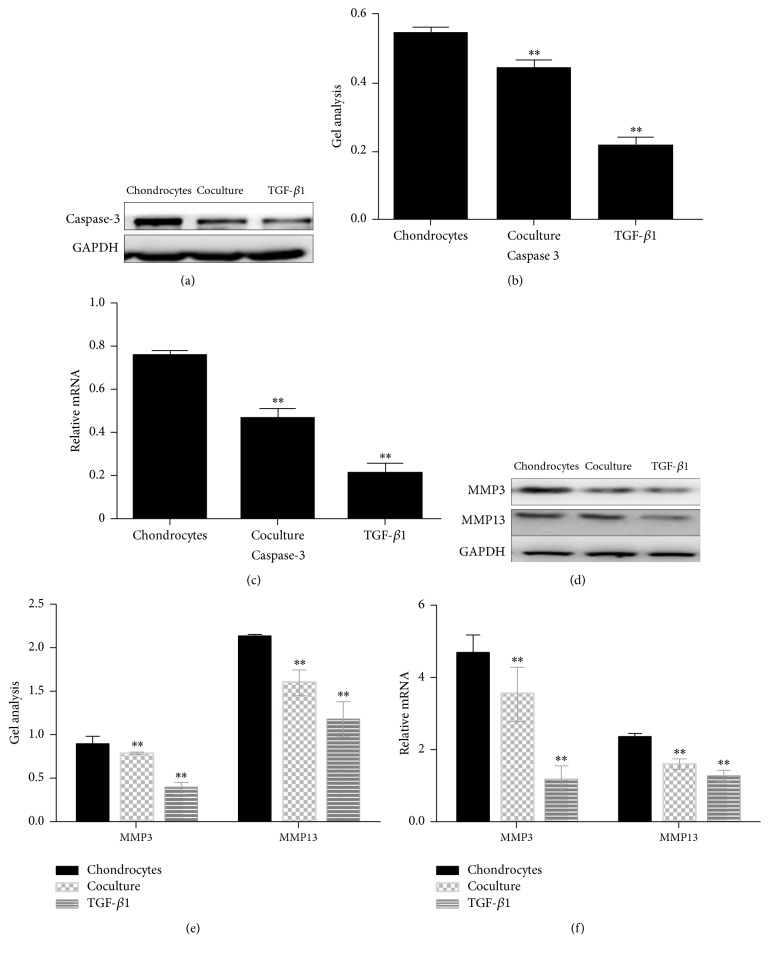
Expression of catabolic markers on chondrocytes cultured with three different conditions for 5 days and determined by western blot ((a), (b), (d), and (e)) and real-time PCR ((c), (f)). Caspase-3 protein was downregulated in chondrocytes when they were cultured with ADSCs cocultured system and TGF-*β*1-containing medium ((a), (b)). The mRNA level of caspase-3 was also decreased in chondrocytes cultured with ADSCs cocultured system and TGF-*β*1-containing medium (c). Furthermore, both MMP3 and MMP13 were suppressed in chondrocytes cultured with ADSCs cocultured system and TGF-*β*1-containing medium tested by western blot ((d), (e)) and real-time PCR (f). Data are shown as mean ± SEM. ^*∗∗*^ *p* < 0.01 versus chondrocytes group.

It has been reported that caspase-3 was a protein which played an important role in the apoptosis of cells [14]. In order to find out how the ADSCs inhibit the apoptosis of chondrocytes, we used western blot and real-time PCR to detect caspase-3 level in chondrocytes culture under three different conditions (Figures 8(a), 8(b), and 8(c)). The results showed that the expression of caspase-3 was inhibited in chondrocytes cocultured with ADSCs group and TGF-*β*1-containing culture medium group (Figures 8(a), 8(b), and 8(c)). Some studies have indicated that some matrix metalloproteinase (MMP) such as MMP3 and MMP13 can damage the chondrocytes [15, 16]. Our results showed that ADSCs reduced the destruction of matrix metalloproteinase in chondrocytes. Similar results were also found in the chondrocytes cultured with TGF-*β*1-containing medium (Figures 8(c), 8(d), and 8(e)).

**Figure 9:**
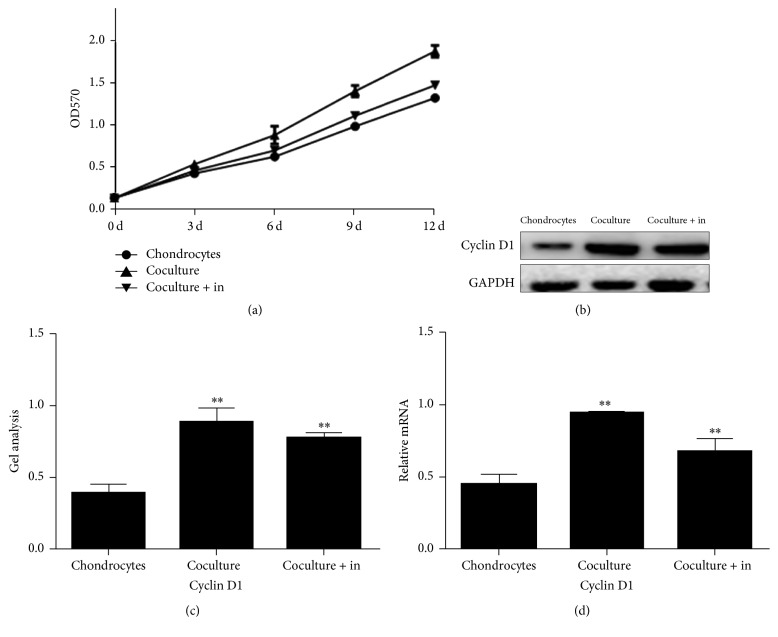
Effect of TGF-*β*1 inhibitor on proliferation of chondrocytes cultured with different conditions for 5 days determined by MTT assay (a), western blot ((b), (c)), and real-time PCR (d). MMT assay indicated that chondrocytes grew much faster in ADSCs coculture system than control medium; however, when TGF-*β*1 inhibitor was added to chondrocytes cocultured with ADSCs system, their proliferation was inhibited. Western blot and real-time PCR demonstrated that both gene expression and protein expression of Cyclin D1 were increased in chondrocytes which were cultured with ADSCs coculture system, but Cyclin D1 was not as high in chondrocytes when they were cultured with TGF-*β*1 inhibitor-containing ADSCs cocultured system ((b), (c), and (d)). Chondrocytes: chondrocytes were cultured in normal medium. Coculture: chondrocytes were cocultured with ADSCs. Cocultured + in: ADSCs cocultured system with TGF-*β*1 inhibitor added. Data are shown as mean ± SEM. ^*∗∗*^ *p* < 0.01 versus control group.

**Figure 10:**
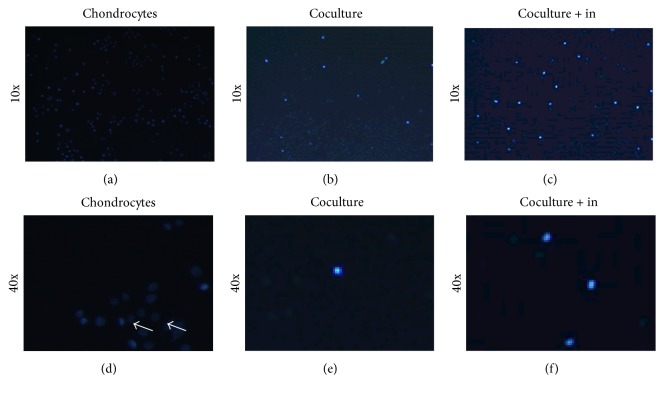
Effect of TGF-*β*1 inhibitor on apoptosis analysis of chondrocytes cultured in three different conditions for 5 days determined by Hoechst 33258 staining. ((a), (d)) Chondrocytes were cultured in normal medium (chondrocytes). ((b), (e)) Chondrocytes were cocultured with ADSCs (coculture). ((c), (f)) Chondrocytes were cultured with ADSCs and TGF-*β*1 inhibitor (coculture + in). Many apoptosis cells were found in chondrocytes cultured in normal medium ((a), (d)); however, ADSCs coculture system inhibited the apoptosis of chondrocytes ((b), (e)). The inhibition effect of ADSCs on apoptosis was blocked by TGF-*β*1 inhibitor ((c), (f)).

However, the promoting effect of ADSCs on chondrocyte proliferation was inhibited by TGF-*β*1 inhibitor. MTT assay showed that TGF-*β*1 inhibitor downregulated the proliferation of chondrocytes when the TGF-*β*1 inhibitor was added in chondrocytes cocultured with ADSCs (Figure 9(a)). Western blot and real-time PCR showed that TGF-*β*1 inhibitors decreased the Cyclin D1 expression which was promoted by ADSCs (Figures 9(b), 9(c), and 9(d)). Hoechst 33258 staining assay showed that many cells with condensed chromatin were observed in chondrocytes group (Figures 10(a) and 10(d)), while the condensed chromatin was reduced in the chondrocytes cocultured with ADSCs group (Figures 10(b) and 10(e)). The addition of TGF-*β*1 inhibitor to chondrocytes cocultured with ADSCs system induced more apoptosis cells (Figures 10(c) and 10(f)) than ADSCs-chondrocytes coculture group (Figures 10(b) and 10(e)). The apoptosis rate in different culture systems was in the following order: control > ADSCs coculture + TGF-*β*1 inhibitor > ADSCs coculture (Figures 10(a)–10(f)).

**Figure 11:**
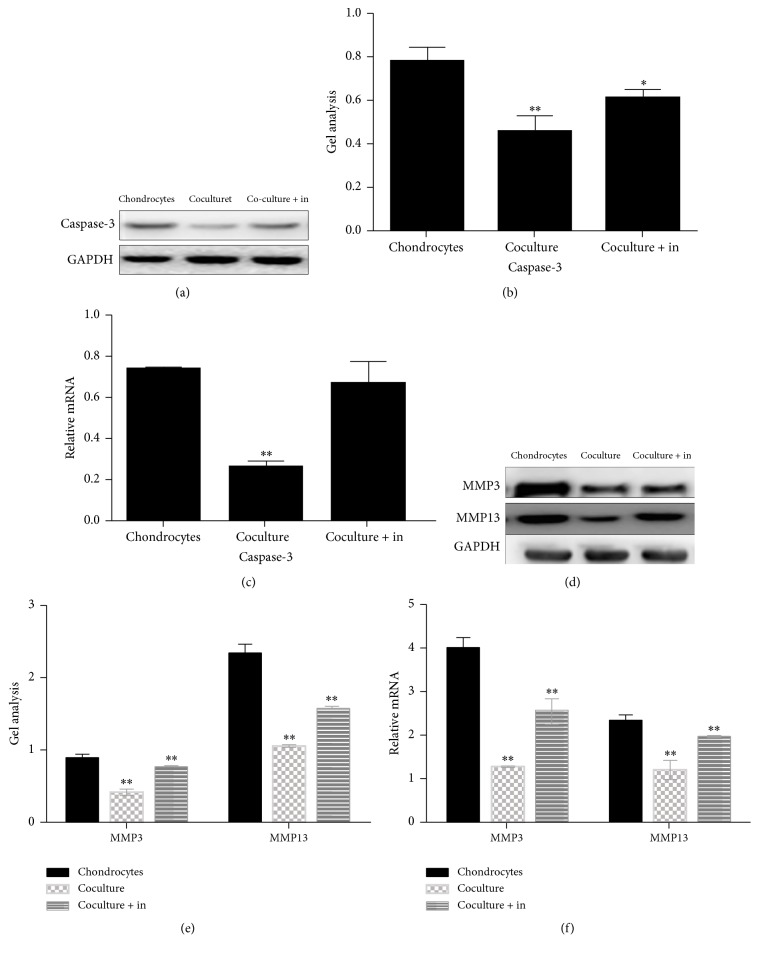
Effect of TGF-*β*1 inhibitor on the expression of catabolic proteins and genes on chondrocytes cultured in different conditions for 5 days determined by western blot ((a), (b), (d), and (e)) and real-time PCR ((c), (f)). Both western blot and real-time PCR showed that some catabolic marker proteins and genes such as caspase-3, MMP3, and MMP13 were downregulated in chondrocytes when they were cultured with ADSCs coculture system and recovered back to the original levels when TGF-*β*1 inhibitor was added in chondrocytes cocultured with ADSCs as tested by western blot ((a), (b), (d), and (e)) and real-time PCR ((c), (f)). Data are shown as mean ± SEM. ^*∗∗*^ *p* < 0.01 versus control group. *∗* means *p* < 0.05 versus chondrocytes.

Next, the apoptosis-associated proteins were further detected by western blot and real-time PCR. Data showed that the expression of caspase-3 was decreased in the chondrocytes cocultured with ADSCs group (Figures 11(a) and 11(b)); however the caspase-3 level was upregulated by adding TGF-*β*1 inhibitor to chondrocytes cocultured with ADSCs group (Figures 11(a), 11(b), and 11(c)). To study whether TGF-*β*1 inhibitor is involved in the production of matrix metalloproteinase such as MMP3 and MMP13, western blot and real-time PCR were performed. Results showed that TGF-*β*1 inhibitors significantly recovered the downregulated of MMP3 and MMP13 by ADSCs to chondrocytes (Figures 11(d), 11(e), and 11(f)).

The above results indicated that ADSCs promoted the proliferation and inhibited the apoptosis of chondrocytes and weakened the protein expression which may damage the growth of chondrocytes, through the activation of TGF-*β*1 pathway.

## 4. Discussion

Trauma, osteoarthritis, and osteomalacia which may cause cartilage injury are very common in clinical practice. The traditional method of articular cartilage repair is mainly through the mobilization of chondrocytes within the healing potential of promoting cartilage healing. However cartilage tissue has no blood supply; it can only rely on the joint fluid to provide nutrition; therefore once the cartilage tissue is damaged it is difficult to repair it. With the development of regenerative medicine, tissue function provides a new way to repair cartilage regeneration.

Tissue engineering technology requires a large number of seed cells. Stem cells derived from adipose tissue have a wide range of sources. ADSCs are easy to obtain with high stem cell content and when taken the damage to the donor is small. At the same time ADSCs have the potential of multidirectional differentiation and can escape immune recognition. In addition, ADSCs are also able to secrete functional cytokines to induce cartilage formation, such as TGF-*β*1, BMP4, and FGF and others, which can promote the regeneration of chondrocytes. Studies have indicated that TGF-*β*1 can induce ADSCs to differentiate into chondrocytes after being transfected into ADSCs. At present, a lot of research has shown that when ADSCs and chondrocytes are cocultured, chondrocytes can prompt the ADSCs to differentiate into chondrocytes. However there are few reports that show whether or not cocultured ADSCs and chondrocytes can promote the regeneration of chondrocytes. TGF-*β*1 has long been known to serve as major regulators in chondrogenesis and osteogenesis [17, 18]. There is also research that had found that TGF-*β*1 always played an essential role in chondrocyte maturation. TGF-*β*1 stimulated chondrocytes proliferation but inhibited chondrocyte differentiation [19]. Recently in order to induce chondrocytes repair of the damaged cartilage several investigations have focused on how to deliver TGF-*β*1 to damaged articular cartilage in vivo [20]. TGF-*β*1 signaling has been found to adjust several proteins such as Cyclin D1 and caspase-3 to promote the growth of chondrocytes [21]. Expressions of type II collagen, SOX9, and PGC-1*α*, all, are prominent components of cartilage [22–24] and can be adjusted by TGF-*β*1. According to the reports, we knew that both ADSCs and TGF-*β*1 can promote the growth of chondrocytes, but currently there were no reports about how ADSCs promote the growth of chondrocytes and if this mechanism has any relationship with TGF-*β*1 signaling when ADSCs are cocultured with chondrocytes.

In our study after extracting adipose-derived stem cells and chondrocytes from New Zealand white rabbits, we used the transwell chambers to coculture ADSCs and chondrocytes (cell ratio of 1 : 1). And we used TGF-*β*1 which was recognized as a promoter of the proliferation of chondrocytes as a positive control [25]. The proliferation of chondrocytes was detected at different time points, and we found that both ADSCs and TGF-*β*1 could promote the proliferation of chondrocytes. In order to investigate how ADSCs promote the proliferation of chondrocytes, we examined the changes of Cyclin D1 which was an important protein in controlling the cell cycle that led to the proliferation of chondrocytes in the experiment [26, 27]. The results showed that Cyclin D1 can be promoted by ADSCs and TGF-*β*1. Then we found that ADSCs could also inhibit the apoptosis of chondrocytes by adjusting the expression of caspase-3, which showed us that ADSCs could promote the proliferation of chondrocytes from another point of view. Some studies have showed that matrix metalloproteinase can damage the regeneration of chondrocytes to some extent [28], so we tested the effect of ADSCs on MMP3 and MMP13. The results confirmed that ADSCs and TGF-*β*1 can inhibit the expression of these two proteins to a certain extent, which can also indicate that ADSCs play a role in promoting the regeneration of cartilage. In the present study, we confirmed that ADSCs can promote the release of GAG, and SOX9, PGC-1*α*, and a series of cartilage regeneration marker proteins [29–32] expression in chondrocytes can be promoted by coculture with ADSCs. This further showed that ADSCs can not only promote the proliferation of cartilage cells, but also have a protective effect on the regeneration of cartilage cells and this effect is similar to that of TGF-*β*1.

In order to confirm the specific mechanism of ADSCs for the role of chondrocytes, we determined the TGF-*β*1 expression in chondrocytes cultured with different conditions. We found that TGF-*β*1 level was increased in the chondrocytes cocultured with ADSCs when compared to chondrocytes only (Figures 6(a), 6(b), and 6(c)). However, when TGF-*β*1 inhibitor was added to the chondrocytes cocultured with ADSCs, the TGF-*β*1 level was significantly decreased (Figures 6(e), 6(f), and 6(g)). Furthermore, with the addition of TGF-*β*1 inhibitors to the cocultured chondrocytes, it was found that TGF-*β*1 inhibitor could inhibit the proliferation of the promotion to chondrocytes by ADSCs, which demonstrated that ADSCs could promote the regeneration of chondrocytes through the TGF-*β*1 pathway.

We confirmed that ADSCs can promote the proliferation and regeneration of chondrocytes through a series of experiments in vitro. This promoting effect is closely related to the TGF-*β*1 pathway. Although we still need animal testing to further confirm our inference, the present study can still provide some new treatment ideas for cartilage damage repair.

## Figures and Tables

**Table 1:** The primer sequences for detection of gene expression.

Name	Forward primer (5′-3′)	Reverse primer (5′-3′)
Cyclin D1	CCGAGGAGCTGCTGCAAATGGAG	GAAATCGTGCGGGGTCATTGCG
Caspase-3	TGTGAGGCGGTTGTAGAAGTT	GCTGCATCGACATCTGTACC
MMP3	AGTTTGCTCAGCCTATCC	GGGTGGAATGTATGTC
MMP13	TGCCCTTATTTTATGTTTCC	TTCCGCTTCCTAGTCAGTTG
TGF-*β*1	CCTGAGTGGCTGTCTTTTGA	GCGCACAATCATGTTGGACA
SOX9	GGTGAACTGGGGGAGGATTG	TCTCGTTGATTTCGCTGCTC
Col2*α*1	CTGGCTCCCAACACTGCCAACG	TCCTTTGGGTTTGCAACGGATTG
PGC-1*α*	ACTATTGAACGCACCTT	CTGGGATGACCGAAGT
GAPDH	CTCTGCTCCTCCTGTTCGAC	GCGCCCAATACGACCAAATC

## References

[1] Madry H., Orth P., Cucchiarini M. (2016). Role of the Subchondral Bone in Articular Cartilage Degeneration and Repair. *Journal of the American Academy of Orthopaedic Surgeons*.

[2] Mak J., Jablonski C. L., Leonard C. A. (2016). Intra-articular injection of synovial mesenchymal stem cells improves cartilage repair in a mouse injury model. *Scientific Reports*.

[3] Reissis D., Tang Q. O., Cooper N. C. (2016). Current clinical evidence for the use of mesenchymal stem cells in articular cartilage repair. *Expert Opinion on Biological Therapy*.

[4] Orth P., Duffner J., Zurakowski D., Cucchiarini M., Madry H. (2016). Small-diameter awls improve articular cartilage repair after microfracture treatment in a translational animal model. *American Journal of Sports Medicine*.

[5] Campbell A. B., Pineda M., Harris J. D., Flanigan D. C. (2016). Return to sport after articular cartilage repair in athletes' knees: a systematic review. *Arthroscopy*.

[6] Richter D. L., Schenck R. C., Wascher D. C., Treme G. (2016). Knee articular cartilage repair and restoration techniques: a review of the literature. *Sports Health*.

[7] Li G., Fu N., Xie J. (2015). Poly(3-hydroxybutyrate-co-4-hydroxybutyrate) based electrospun 3D scaffolds for delivery of autogeneic chondrocytes and adipose-derived stem cells: Evaluation of cartilage defects in rabbit. *Journal of Biomedical Nanotechnology*.

[8] Suganuma S., Tada K., Hayashi K. (2013). Uncultured adipose-derived regenerative cells promote peripheral nerve regeneration. *Journal of Orthopaedic Science*.

[9] Zuk P. A., Zhu M., Mizuno H. (2001). Multilineage cells from human adipose tissue: implications for cell-based therapies. *Tissue Engineering*.

[10] Gosset M., Berenbaum F., Thirion S., Jacques C. (2008). Primary culture and phenotyping of murine chondrocytes. *Nature Protocols*.

[11] Yuan H., Zhang J., Zhang R. (2013). experimental study on adipose-derived stem cells transfected by bone morphogenetic protein 14 co-culture with chondrocytes. *Chinese Journal of Reparative and Reconstructive Surgery*.

[12] Bjornsson S. (1993). Simultaneous preparation and quantitation of proteoglycans by precipitation with Alcian blue. *Analytical Biochemistry*.

[13] Guo B., Zhang Y., Hui Q., Wang H., Tao K. (2016). Naringin suppresses the metabolism of A375 cells by inhibiting the phosphorylation of c-Src. *Tumor Biology*.

[14] Bao G., Xu L., Xu X. (2016). SGTB promotes the caspase-dependent apoptosis in chondrocytes of osteoarthritis. *Inflammation*.

[15] Ji J.-B., Li X.-F., Liu L., Wang G.-Z., Yan X.-F. (2015). Effect of low intensity pulsed ultrasound on expression of TIMP-2 in serum and expression of mmp-13 in articular cartilage of rabbits with knee osteoarthritis. *Asian Pacific Journal of Tropical Medicine*.

[16] Fukui T., Tenborg E., Yik J. H. N., Haudenschild D. R. (2015). In-vitro and in-vivo imaging of MMP activity in cartilage and joint injury. *Biochemical and Biophysical Research Communications*.

[17] Green J. D., Tollemar V., Dougherty M. (2015). Multifaceted signaling regulators of chondrogenesis: implications in cartilage regeneration and tissue engineering. *Genes & Diseases*.

[18] Joyce M. E., Roberts A. B., Sporn M. B., Bolander M. E. (1990). Transforming growth factor-*β* and the initiation of chondrogenesis and osteogenesis in the rat femur. *Journal of Cell Biology*.

[19] Cleary M. A., van Osch G. J. V. M., Brama P. A., Hellingman C. A., Narcisi R. (2015). FGF, TGF*β* and Wnt crosstalk: embryonic to in vitro cartilage development from mesenchymal stem cells. *Journal of Tissue Engineering and Regenerative Medicine*.

[20] Madry H., Rey-Rico A., Venkatesan J. K., Johnstone B., Cucchiarini M. (2014). Transforming growth factor beta-releasing scaffolds for cartilage tissue engineering. *Tissue Engineering-Part B: Reviews*.

[21] Li T.-F., Chen D., Wu Q. (2006). Transforming growth factor-*β* stimulates cyclin D1 expression through activation of *β*-catenin signaling in chondrocytes. *The Journal of Biological Chemistry*.

[22] Buma P., Pieper J. S., van Tienen T. (2003). Cross-linked type I and type II collagenous matrices for the repair of full-thickness articular cartilage defects—a study in rabbits. *Biomaterials*.

[23] James A. W., Xu Y., Lee J. K., Wang R., Longaker M. T. (2009). Differential effects of TGF-*β*1 and TGF-*β*3 on chondrogenesis in posterofrontal cranial suture-derived mesenchymal cells in vitro. *Plastic and Reconstructive Surgery*.

[24] Lee Y. J., Kong M. H., Song K. Y., Lee K. H., Heo S. H. (2008). The relation between Sox9, TGF-*β*1, and proteoglycan in human intervertebral disc cells. *Journal of Korean Neurosurgical Society*.

[25] Almeida H. V., Cunniffe G. M., Vinardell T., Buckley C. T., O'Brien F. J., Kelly D. J. (2015). Coupling freshly isolated CD44^+^ infrapatellar fat pad-derived stromal cells with a TGF-*β*3 eluting cartilage ECM-derived scaffold as a single-stage strategy for promoting chondrogenesis. *Advanced Healthcare Materials*.

[26] He C.-X., Zhang T.-Y., Miao P.-H. (2012). TGF-*α*1 gene-engineered mesenchymal stem cells induce rat cartilage regeneration using nonviral gene vector. *Biotechnology and Applied Biochemistry*.

[27] Mello M. A., Tuan R. S. (2006). Effects of TGF-*β*1 and Triiodothyronine on cartilage maturation: in vitro analysis using long-term high-density micromass cultures of chick embryonic limb mesenchymal cells. *Journal of Orthopaedic Research*.

[28] Patil A., Sable R., Kothari R. (2012). Genetic expression of MMP-Matrix-Mettalo-Proteinases (MMP-1 and MMP-13) as a function of anterior mandibular repositioning appliance on the growth of mandibular condylar cartilage with and without administration of Insulin like growth factor (IGF-1) and transforming growth factor-B (TGF-*β*). *The Angle Orthodontist*.

[29] Ustun Yaylaci S., Sardan Ekiz M., Arslan E. (2016). Supramolecular GAG-like self-assembled glycopeptide nanofibers induce chondrogenesis and cartilage regeneration. *Biomacromolecules*.

[30] Chang T., Xie J., Li H., Li D., Liu P., Hu Y. (2016). MicroRNA-30a promotes extracellular matrix degradation in articular cartilage via downregulation of Sox9. *Cell Proliferation*.

[31] Diederichs S., Gabler J., Autenrieth J. (2016). Differential regulation of SOX9 protein during chondrogenesis of induced pluripotent stem cells versus mesenchymal stromal cells: a shortcoming for cartilage formation. *Stem Cells and Development*.

[32] Frisch J., Rey-Rico A., Venkatesan J. K., Schmitt G., Madry H., Cucchiarini M. (2015). rAAV-mediated overexpression of sox9, TGF-*β* and IGF-I in minipig bone marrow aspirates to enhance the chondrogenic processes for cartilage repair. *Gene Therapy*.

